# Modulation of Early Inflammatory Response by Different Balanced and Non-Balanced Colloids and Crystalloids in a Rodent Model of Endotoxemia

**DOI:** 10.1371/journal.pone.0093863

**Published:** 2014-04-07

**Authors:** Stefanie Voigtsberger, Martin Urner, Melanie Hasler, Birgit Roth Z'Graggen, Christa Booy, Donat R. Spahn, Beatrice Beck-Schimmer

**Affiliations:** 1 Institute of Anesthesiology, University Hospital Zurich, Zurich, Switzerland,; 2 Institute of Physiology and Zurich Center for Integrative Human Physiology, University of Zurich, Zurich, Switzerland; University of Illinois at Chicago, United States of America

## Abstract

The use of hydroxyethyl starch (HES) in sepsis has been shown to increase mortality and acute kidney injury. However, the knowledge of the exact mechanism by which several fluids, especially starch preparations may impair end-organ function particularly in the kidney, is still missing. The aim of this study was to measure the influence of different crystalloid and colloid fluid compositions on the inflammatory response in the kidney, the liver and the lung using a rodent model of acute endotoxemia. Rats were anesthetized and mechanically ventilated. Lipopolysaccharide (5 mg/kg) was administered intravenously. After one hour crystalloids [lactate-buffered (RLac) or acetate-buffered (RAc)] were infused i.v. (30 ml/kg) in all groups. At 2 hours rats either received different crystalloids (75 ml/kg of RLac or RAc) or colloids (25 ml/kg of HES in saline or HES in RAc or gelatin in saline). Expression of messenger RNA for cytokine-induced neutrophil chemoattractant-1 (CINC-1), monocyte chemotactic protein-1 (MCP-1), necrosis factor α (TNFα) and intercellular adhesion molecule 1 (ICAM-1) was assessed in kidney, liver and lung tissue by real-time PCR after 4 hours. The use of acetate-buffered solutions was associated with a significantly higher expression of CINC-1 and TNFα mRNA in the liver, in the kidney and in the lung. Only marginal effects of gelatin and hydroxyethyl starch on mRNA expression of inflammatory mediators were observed. The study provides evidence that the type of buffering agent of different colloidal and crystalloid solutions might be a crucial factor determining the extent of early end-organ inflammatory response in sepsis.

## Introduction

Acute kidney injury (AKI) is a common complication in sepsis. It has a prevalence of 25% in severe sepsis and up to 50% in septic shock [1]. The mortality of patients with AKI in sepsis can be up to 75% [2]. The pathogenesis of sepsis-induced AKI is multifactorial. Hemodynamic factors like hypovolaemia and hypoxaemia as well as non-haemodynamic mechanisms as a combination of immunologic, toxic and inflammatory factors have been shown to affect the microvasculature and the tubular cells resulting in organ dysfunction [3].

Immediate fluid resuscitation is a main strategy in the early-goal directed therapy (EGDT) of sepsis and septic shock and has been shown to improve outcome [4], [5]. In the past, different type of fluids, colloids as well as crystalloids, have been used to perform fluid resuscitation. The new sepsis guidelines, however, clearly recommend to use crystalloids for initial fluid resuscitation and to avoid hydroxyethyl starch (HES) preparations [6]. Particularly with regard to sepsis the potential of HES to impair renal function has been discussed extensively [7], [8]. The effects on renal function seem to depend at least in part on the used HES preparation: HES products with a higher molecular weight have been shown to impair kidney function. According to the work from Schortgen et al. 6% HES 200/0.60–0.66 is an independent risk factor for AKI in patients with severe sepsis or septic shock [7]. In the VISEP study (Volume Substitution and Insulin Therapy in Severe Sepsis) the use of 10% HES 200/0.5 was associated with a higher incidence of AKI and renal replacement therapy compared to Ringer's Lactate [9]. Sparse data is available for the effect of 6% HES 130 on renal function in sepsis. The CRYSTMAS study recently demonstrated no differences in mortality, coagulation and the occurrence of AKI between 6% HES 130 and normal saline in patient with a severe sepsis [10]. However, the 6S-Trial showed increased risk of death at day 90 and a higher incidence for renal replacement therapy in patients with severe sepsis treated with 6% HES 130/0.42 in acetate (HES-RAc) compared to Ringer's Acetate (RAc) [11]. As recently pointed out by Soni et al., there is currently still a lack of knowledge on the exact mechanism by which HES may impair kidney function despite the existence of a plethora of clinical and experimental studies [12]. A modulation of the inflammatory response caused by the different type of fluids has been suggested to play a key role. In a previous study, we demonstrated in an *in vitro* model of human proximal tubular epithelial cells (HK-2) that HES products modulate cell injury upon inflammatory stimulation with tumor necrosis factor α (TNFα) with a more pronounced effect for HES 200/0.5 than for HES 130/0.42 [13]. Additionally, since the introduction of “plasma-adapted” fluids during the recent years the discussion on optimal fluid resucitation has been extended to the question of the buffering agent. The main purpose of the introduction of balanced “plasma-adapted” solutions was the reduction of electrolyte- and acid base-disturbances like hyperchloraemic acidosis caused by the use of large volumes of saline-based fluids [14]. However, the clinical relevance of hyperchloremic acidosis is currently unclear [14].

In the present study we used a rodent model of lipopolysaccharides (LPS)-induced sepsis to compare the effects of different colloidal and crystalloid solutions shortly after their administration on (1) inflammatory response in vital organs (kidney, liver, lung) (2) renal injury (3) haemodynamics and (4) acid-base status. As HES 200/0.5 has already been removed from our clinical routine, we focused on the currently used colloids: 6% HES 130/0.42 and 4% gelatin. Moreover, we were interested in the question whether the solvent (saline-based or balanced  =  “plasma-adapted”) has an influence on the inflammatory response. Since the usage of the newer plasma-adapted solutions containing acetate/malate as a buffer we were wondering if the different organic anions as ingredients of balanced solutions (acetate/malate or lactate) may have an influence on inflammatory response. Therefore 5 groups were compared: 6% HES 130/0.42 in 0.9% NaCl (HES-NaCl), 6% HES/0.4 in RAc (HES-RAc), 4% gelatin in 0.9% NaCl (Gel-NaCl), Ringer's Lactate (RLac) and Ringer's Acetate (RAc).

## Materials and Methods

### Ethics statement

The protocol of this study was approved by the local animal care and use committee (Kantonales Veterinäramt, Zürich; Permit Number: 132/2007). All surgery was performed under sodium thiopenthal anesthesia. The experiments were performed under sedation with sevoflurane, and all efforts were made to minimize suffering.

### Animal preparation

Pathogen-free, male Wistar rats weighing 350–500 g (Charles River, Germany) were used in the experiments. The rats were housed in standard cages at 22±1°C temperature under a 12/12-hour light/dark regimen. Food and water were supplied ad libitum.

For initial anesthesia sodium thiopenthal (100 mg/kg; Pentothal, Swissmedic, Ospedalia AG, Switzerland) was given intraperitoneally. For measurement of mean arterial blood pressure (MAP) the left carotid artery was cannulated with a sterile polyethylene catheter and connected to a pressure transducer (Spacelabs, Hertford, United Kingdom). Measurement of MAP was monitored continuously. Arterial blood gases were analysed at time points 0, 2 and 4 hours. Fluid resuscitation was realized by cannulation of the tail vein with a sterile 22 GA catheter (BD Insyte Becton Dickinson S.A., Madrid, Spain).

Experiments were performed under sedation with sevoflurane. Therefore rats were tracheotomised and intubated with a sterile metal cannula. Mechanical ventilation was performed in a pressure controlled modus with a Servo ventilator 300 (Solna, Sweden). (Peak inspiratory pressure: 15 cm H_2_O, positive end-expiratory pressure: 2 cm H_2_O, fractional inspired oxygen fraction: 1.0, I/E ratio: 1∶2, breathing frequency: 30 breaths min^−1^. Body temperature was monitored continuously and maintained at 37°C by a heating lamp.

Sevoflurane was administered via the AnaConDa system (Anaesthetic Conserving Device, Sedana Medical, Uppsala, Sweden). For measurement of the expiratory concentration of sevoflurane and CO_2_ the multigas analyzer (VEO Multigas Monitor, PHASEIN medical technologies, Danderyd, Sweden) was used. For sedation a concentration of 1–2 Vol.%, respectively 0.5–1 minimal alveolar concentration (MAC) of sevoflurane was administered.

### Experimental design

Five different fluids were tested, all purchased from B. Braun, Melsungen, Germany: ***1. Saline-based colloid***: 6% HES 130/0.42 prepared in a saline solution (Na^+^ 154 mmol/l, Cl^−^ 154 mmol/l; Venofundin), ***[HES-NaCl]***; ***2. Acetate-buffered colloid***: 6% HES 130/0.42 prepared in a plasma adapted solution (Na^+^ 140 mmol/l, Cl^−^ 118 mmol/l, K^+^ 4 mmol/l, Mg^2+^ 1 mmol/l, Ca^2+^ 2.5 mmol/l, acetate 24 mmol/l, malate 5 mmol/l; Tetraspan), ***[HES-RAc]***; ***3. Saline-based colloid***: 4% gelatin prepared in a saline solution (Na^+^ 154 mmol/l, Cl^−^ 154 mmol/l; Physiogel), ***[Gel-NaCl]***; ***4. Lactate-buffered crystalloid***: Ringer's Lactate (Na^+^ 130.5 mmol/l, K^+^ 5.36 mmol/l, Ca^2+^ 1.84 mmol/l, Cl^−^ 111.7 mmol/l, lactate 27.8 mmol/l; Ringer's Lactate), ***[RLac]***; ***5. Acetate-buffered crystalloid***: Ringer's Acetate (Na^+^ 140 mmol/l, Cl^−^ 127 mmol/l, K^+^ 4 mmol/l, Mg^2+^ 1 mmol/l, Ca^2+^ 2.5 mmol/l, acetate 24 mmol/l, malate 5 mmol/l; Ringerfundin), ***[RAc]***. The composition of the different fluids is summarized in **Table 1**.

**Table 1:** Composition of fluids.

	RLac	RAc	HES-NaCl	HES-RAc	GEL-NaCl
**Na^+^, mmol/L**	130.5	140	154	140	154
**Cl^−^, mmol/L**	111.7	127	154	118	120
**K^+^, mmol/L**	5.36	4	-	4	-
**Mg^2+^, mmol/L**	-	1	-	1	-
**Ca^2+^, mmol/L**	1.84	2.5	-	2.5	-
**Acetate, mmol/L**	-	24	-	24	-
**Malate, mmol/L**	-	5	-	5	-
**Lactate, mmol/L**	27.8	-	-		-
**pH**	5.0–7.0	4.6–5.4	4.0–5.5	5.6–6.4	7.1–7.7
**Osmolarity, mosm/L**	278	304	308	288	274

After the preparation rats were randomly assigned to one of the following 10 groups: 1. ***HES-NaCl*** [+ lipopolysaccharide (LPS)] or [+ phosphate buffered saline (PBS)], 2. ***HES-RAc*** (+ LPS) or (+PBS), 3. ***Gel-NaCl*** (+ LPS) or (+ PBS), 4. ***RLac*** (+ LPS) or (+ PBS), 5. ***RAc*** (+ LPS) or (+ PBS). For all LPS groups 6 rats were assigned to each group. For all PBS groups 3 rats were assigned to each group.

**Figure 1:**
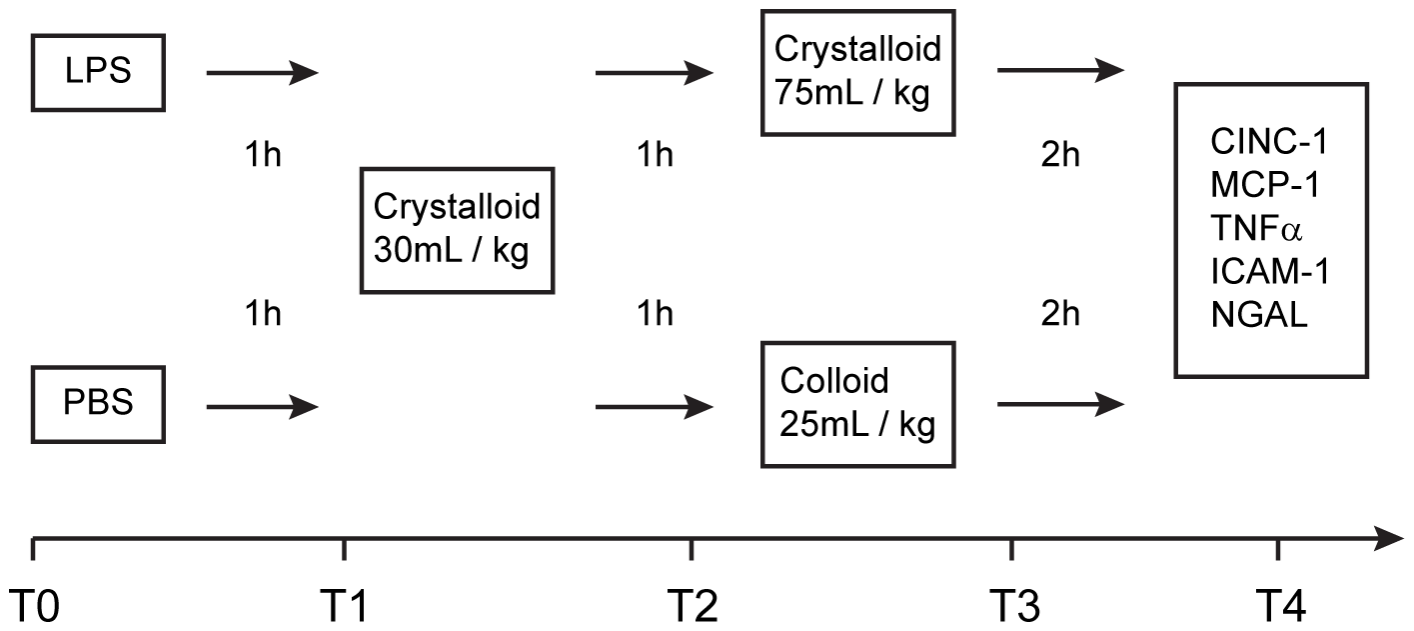
Experimental setting. After the preparation animals were randomized to one of the following groups: 1. ***HES-NaCl*** (+ LPS) **or** (+ PBS), 2. ***HES-RAc*** (+ LPS) **or** (+PBS), 3. ***Gel-NaCl*** (+ LPS) **or** (+ PBS), 4. ***RLac*** (+ LPS) **or** (+ PBS), 5. ***RAc*** (+ LPS) **or** (+ PBS). **[T0]**: Rats received either lipopolysaccharides (LPS) (5 mg/kg in 500 μl PBS) or phosphate-buffered saline (PBS) (500 μl) intravenously. **[T1]**: After 1 hour all groups received (***first volume bolus***) with crystalloids at a volume of 30 ml/kg: Ringer's Lactate was used in all saline-based colloid groups (***HES-NaCl, Gel-NaCl*** *,*) and in the Ringer's Lactate group itself (***RLac***). Ringer's Acetate was used in all acetate-balanced groups (***HES-RAc, RAc***). **[T2]**: After two hours fluid resuscitation was continued according to the groups (***second volume bolus***): rats received either crystalloids at a volume of 75 ml/kg according to their group (***RLac, RAc***) or colloids at a volume of 25 ml/kg according to their group (***HES-NaCl, Gel-NaCl, HES-RAc***). **[T4]**: After 4 hours animals were euthanized; kidneys, lungs and liver were harvested and shock frozen for later mRNA preparation

The experimental setting is summarized in **Figure 1**. In the LPS group rats were intravenously instilled with *Escherichia coli*–LPS (serotype 055:B5; Sigma Aldrich, Buchs, Switzerland) at a dose of 5 mg/kg in 500 μl PBS. The control groups only received 500 μl PBS intravenously applied. One hour after the application of LPS or PBS, fluid resuscitation was started with crystalloids at a volume of 30 ml/kg in all groups (***first volume bolus***) to avoid a drop of blood pressure: Ringer's Lactate was used in all saline-based colloid groups (***HES-NaCl, Gel-NaCl*** *,*) and in the Ringer's Lactate group itself (***RLac***). Ringer's Acetate was used in all acetate-balanced groups (***HES-RAc, RAc***). Two hours after application of LPS or PBS fluid resuscitation was continued according to the groups (***second volume bolus***): rats received either crystalloids at a volume of 75 ml/kg according to their group (***RLac, RAc***) or colloids at a volume of 25 ml/kg according to their group (***HES-NaCl, Gel-NaCl, HES-RAc***).

### Preparation and analysis of samples

Four hours after the application of LPS/PBS the animals were euthanized. Blood was taken by puncture of the inferior vena cava. Urine was taken by puncture of the urinary bladder. The collected fluids were centrifuged at 4°C (1500 g, 10 min.) Aliquots of the supernatant were frozen at −20°C. For tissue analysis kidney, liver and lung were harvested, immediately snap-frozen in liquid nitrogen and stored at −80°C for isolation of RNA.

### RNA extraction and real-time PCR for cytokine-induced neutrophile chemoattractant (CINC-1), monocyte chemotactic protein 1 (MCP-1), tumor necrosis factor α (TNFα), intercellular adhesion molecule 1 (ICAM-1) and neutrophil gelatinase-associated lipocalin (NGAL)

Total RNA was isolated from kidney, liver and lung tissue using the RNeasy Mini Kit (Qiagen, Basel, Switzerland) according to the manufacturer's protocol. Tissue was lysed in the provided buffer and subsequently loaded on RNeasy mini spin columns. RNA was eluted with RNAse-free water. Total amounts and purity of RNA were determined by absorbance at 260 nm and the 260/280 nm absorbance-ratio, respectively.

Reverse transcription was performed with 0.5 μg total RNA at 25°C for 10 min, 37°C for 120 min and 85°C for 5 min. Random primers and multiscreen reverse transcriptase were used for cDNA synthesis.

Real time quantitative PCR was performed on a GeneAmp 5700 system (ABI Applied Biosystems, Life Technologies). Specific primers (Microsynth, Balgach, Switzerland) and labeled probes (Roche Probe Library, Basel, Switzerland) were designed for MCP-1, CINC-1, NGAL and 18S. The FastStart Universal Probe Master PCR Mix (Roche, Basel, Switzerland) was used for the assays in a final reaction volume of 15 μl. All primers and probes used in the experiments are presented in **Table S1**. Each experimental PCR run was performed in duplicate with simultaneous assays for controls with no template. For quantitation of gene expression the comparative Ct method was used as described by Livak [15]. The Ct values of samples (LPS-groups) and controls (PBS-groups) were normalized to the housekeeping gene (18S) and calculated as 2^−ΔΔCt^, where ΔΔC_t_  =  ΔC_t,samples_ – ΔC_t,controls_.

### Enzyme-linked immunosorbent assay (ELISA) for neutrophil gelatinase-associated lipocalin (NGAL)

ELISAs were performed according to the manufacture's protocol assessing NGAL (R&D Systems Europe Ltd., Abingdon, United Kingdom) in urine. The detection range was 78.125–5000 ng/ml for NGAL.

### Statistical analysis

Statistical analyses were performed with OriginPro 8G (Origin Lab, Northampton, MA) and SPSS 20 (SPSS Inc., Chicago, IL). Boxplot figures show medians and quartiles. Whiskers represent 5% and 95% confidence intervals, * represent 1% and 99% confidence intervals. Linear regression was used to assess the effects on inflammatory expression in liver, kidney, and lung tissue. Changes in mRNA expression are indicated as fold-difference to animals solely receiving Ringer's Lactate (reference group). Influences on mRNA expression and NGAL protein concentrations (dependent variables, n = 45) were calculated using the following dummy-coded, independent variables: 1) “LPS”: animals stimulated with LPS, 2) “Acetate-buffered solution”: animals receiving fluids containing acetate as buffering agent (HES-RAc or RAc), 3) “Hydroxyethyl starch”: animals receiving fluids containing HES independent from the type of buffering agent (HES-RAc or HES-NaCl), 4) “Gelatin”: animals receiving gelatin (Gel-NaCl). Blood gas analysis and MAP results were analysed with linear regression using the same independent variables as described above. Additionally, the changes in MAP/blood gas results over the time of experimental procedure were addressed introducing time as an ordinal independent predictor (therefore 3×45 = 135 dependent variables). P-values of 0.05 or less were considered statistically significant.

## Results

### 1. Inflammation

#### Inflammatory mediator expression in the kidney

**Figure 2:**
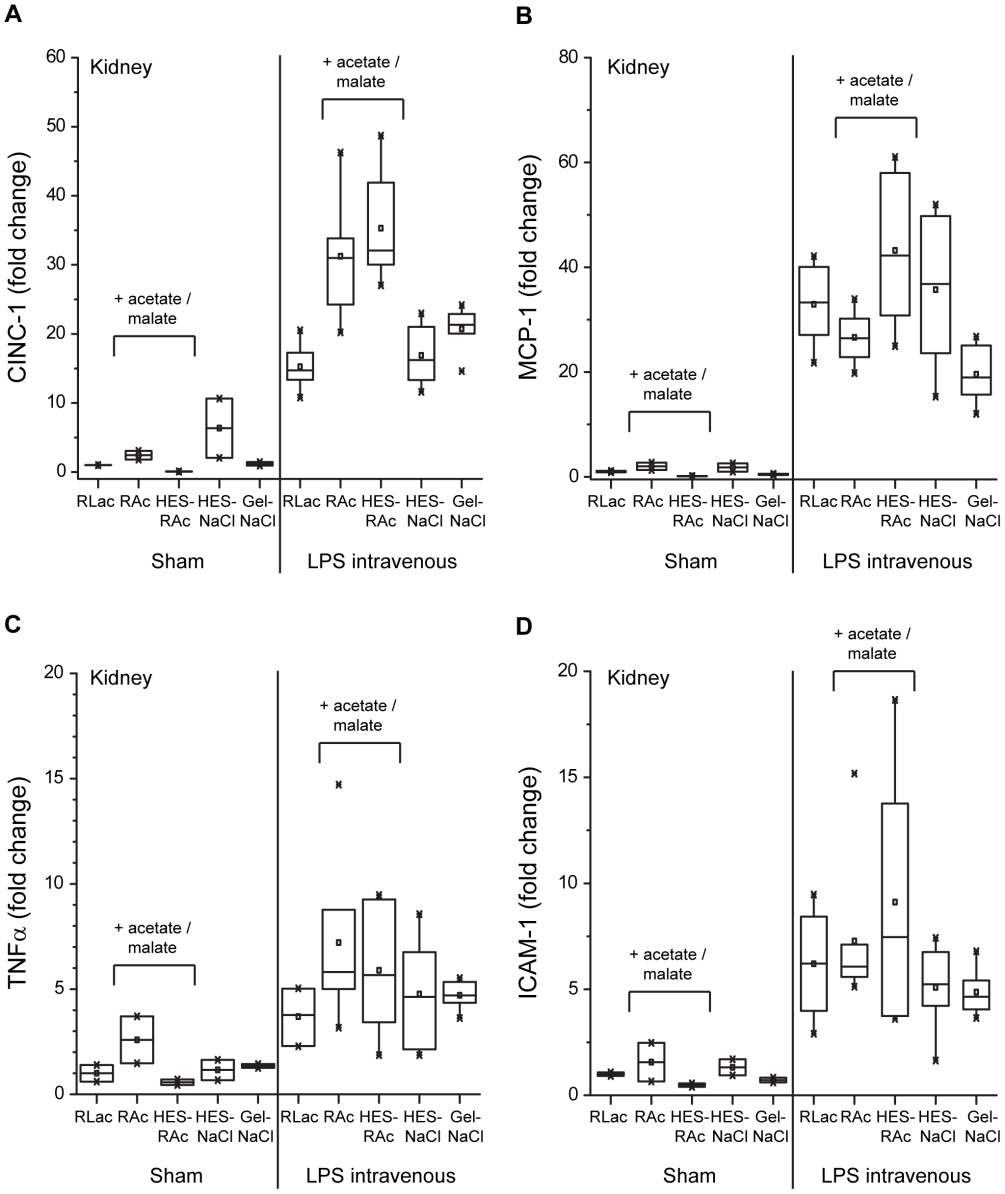
Inflammatory mediator expression in kidney tissue. Evaluation of cytokine-induced neutrophil chemoattractant-1 (CINC-1; A), monocyte chemoattractant protein-1 (MCP-1; B), tumor necrosis factor α (TNFα; C) and intercellular adhesion molecule 1 (ICAM-1; D) messenger RNA expression in kidney tissue. Kidney tissue was collected after 4 hours from the 10 study groups: SHAM (+PBS) or LPS intravenous (+LPS): RLac, HES-NaCl, RAc, HES-RAc, Gel-NaCl. Specific real-time polymerase chain reactions were performed on random transcribed complementary DNA. Values are illustrated as fold change in relation to the RLac group ( = 1.0).

Injection of LPS provoked an increased mRNA expression of CINC-1 (22-fold), of MCP-1 (31-fold), of TNFα (4-fold), and of ICAM-1 (6-fold) in the kidneys (**Figure 2A–D** **,** **Table 2**). Independent of the buffering agent, HES as solution ingredient was not found to have a significant influence on CINC-1, MCP-1, TNFα and ICAM-1 mRNA expression. No effect of gelatin on inflammatory mediator expression was observed. A significant higher expression of CINC-1 (12-fold), of TNFα (2-fold), and of ICAM-1 (2-fold) was found in animals which received acetate-buffered solutions (RAc or HES-RAc).

**Table 2:** Changes in inflammatory mediator expression of the kidneys.

	LPS	HES	Gelatin	Acetate buffer	R^2^
CINC-1, n-fold	**22 (17, 27)^a^**	2 (−2, 7)	4 (−2, 10)	**12 (8, 17)^a^**	0.753
MCP-1, n-fold	**31 (23, 38)^a^**	6 (−1, 14)	−8 (−18, 2)	1 (−6, 8)	0.693
TNF-α, n-fold	**4 (2, 6)^a^**	0 (−2, 1)	0 (−2, 3)	**2 (0, 4)^c^**	0.422
ICAM-1, n-fold	**5 (3, 8)^a^**	0 (−2, 2)	0 (−4, 2)	**2 (0, 4)^a^**	0.440

The table shows B coefficients (95% confidence intervals) of the linear regression. Animals which received solely Ringers' lactate were used as reference group. mRNA expression is expressed as a fold-difference relative to the reference group. The different fluid ingredients (HES, gelatin, and acetate buffer) were entered as binary independent predictors in the regression model. Significance: ^a^p≤0.001, ^b^p≤0.01, ^c^p<0.05; CINC-1  =  cytokine-induced neutrophil chemoattractant-1; MCP-1  =  monocyte chemoattractant protein-1; TNFα  =  tumor necrosis factor α; ICAM-1  =  intercellular adhesion molecule-1.

#### Inflammatory mediator expression in the liver

**Figure 3:**
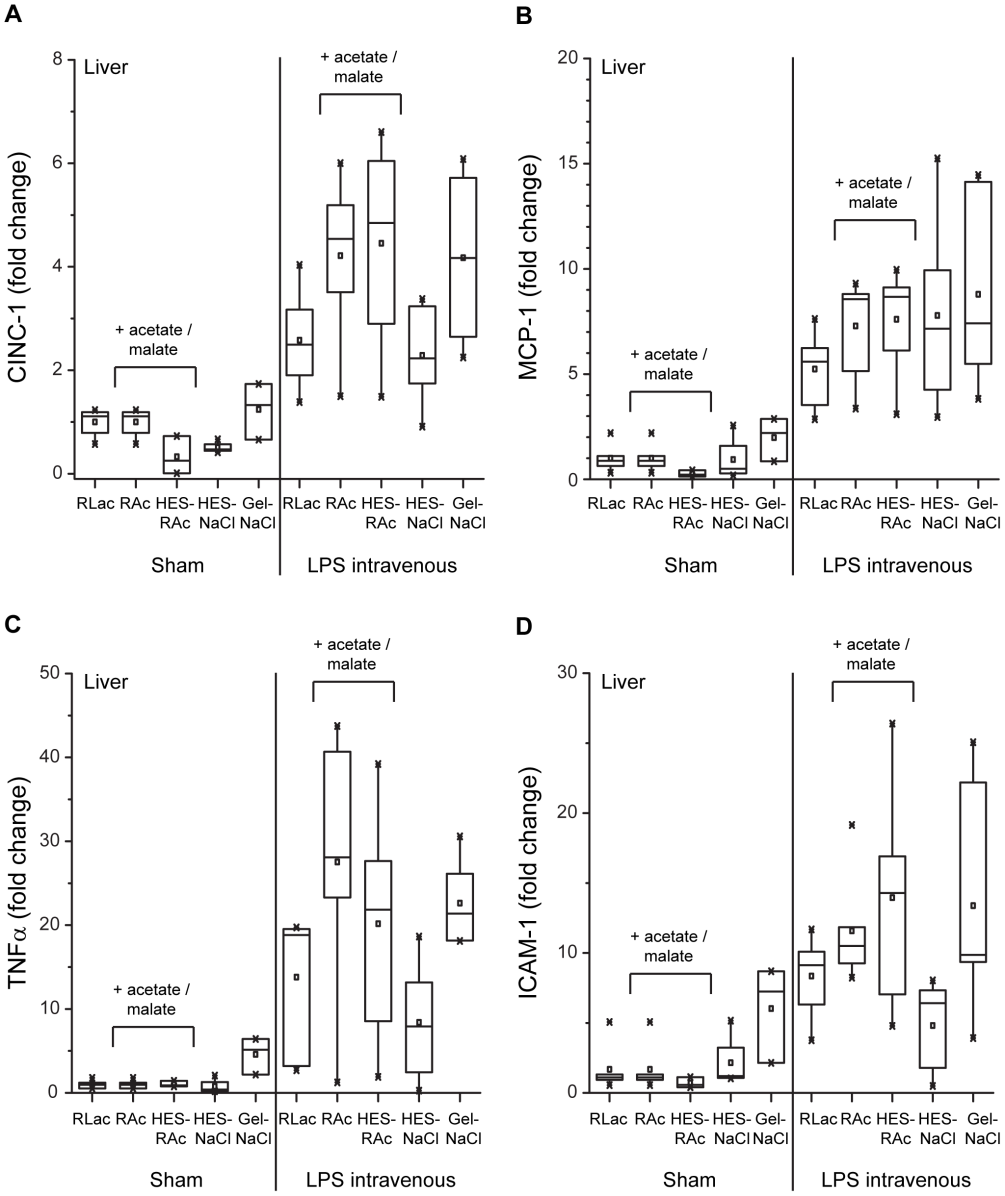
Inflammatory mediator expression in liver tissue. Evaluation of cytokine-induced neutrophil chemoattractant-1 (CINC-1; A), monocyte chemoattractant protein-1 (MCP-1; B), tumor necrosis factor α (TNFα; C) and intercellular adhesion molecule 1 (ICAM-1; D) messenger RNA expression in liver tissue. Liver tissue was collected after 4 hours from the 10 study groups: SHAM (+PBS) or LPS intravenous (+LPS): RLac, HES-NaCl, RAc, HES-RAc, Gel-NaCl. Specific real-time polymerase chain reactions were performed on random transcribed complementary DNA. Values are illustrated as fold change in relation to the RLac group ( = 1.0).

As illustrated in **Figure 3A–D** and **Table 3**, LPS induced an increased mRNA expression of CINC-1 (3-fold), of MCP-1 (6-fold), of TNFα (16-fold), and of ICAM-1 (8-fold) also in liver tissue. No influence of HES on inflammatory mediator expression was found. No effects on MCP-1 or TNFα expression, but marginally elevated CINC-1 and ICAM-1 levels were observed in LPS-animals which received gelatin (**Table 3**). A higher expression of CINC-1 (1-fold), TNFα (10-fold), and ICAM-1 (4-fold) was found in animals which had been given acetate-buffered solutions. This was not observed for MCP-1.

**Table 3:** Changes in inflammatory mediator expression of the liver.

	LPS	HES	Gelatin	Acetate buffer	R^2^
CINC-1, n-fold	**3 (2, 3)^a^**	0 (0, 1)	**1 (0, 2)^c^**	**1 (0, 2)^c^**	0.605
MCP-1, n-fold	**6 (4, 8)^a^**	0 (−2, 2)	2 (0, 4)	1 (−1, 3)	0.584
TNF-α, n-fold	**16 (11, 21)^a^**	1 (−7, 5)	6 (1, 13)	**10 (4, 16)^a^**	0.606
ICAM-1, n-fold	**8 (5, 11)^a^**	1 (−3, 5)	**6 (2, 10)^b^**	**4 (0, 7)^c^**	0.517

The table shows B coefficients (95% confidence intervals) of the linear regression. Animals which received solely Ringers' lactate were used as reference group. mRNA expression is expressed as a fold-difference relative to the reference group. The different fluid ingredients (HES, gelatin, and acetate buffer) were entered as binary independent predictors in the regression model. Significance: ^a^p≤0.001, ^b^p≤0.01, ^c^p<0.05; CINC-1  =  cytokine-induced neutrophil chemoattractant-1; MCP-1  =  monocyte chemoattractant protein-1; TNFα  =  tumor necrosis factor α; ICAM-1  =  intercellular adhesion molecule-1.

#### Inflammatory mediator expression in the lung

**Figure 4:**
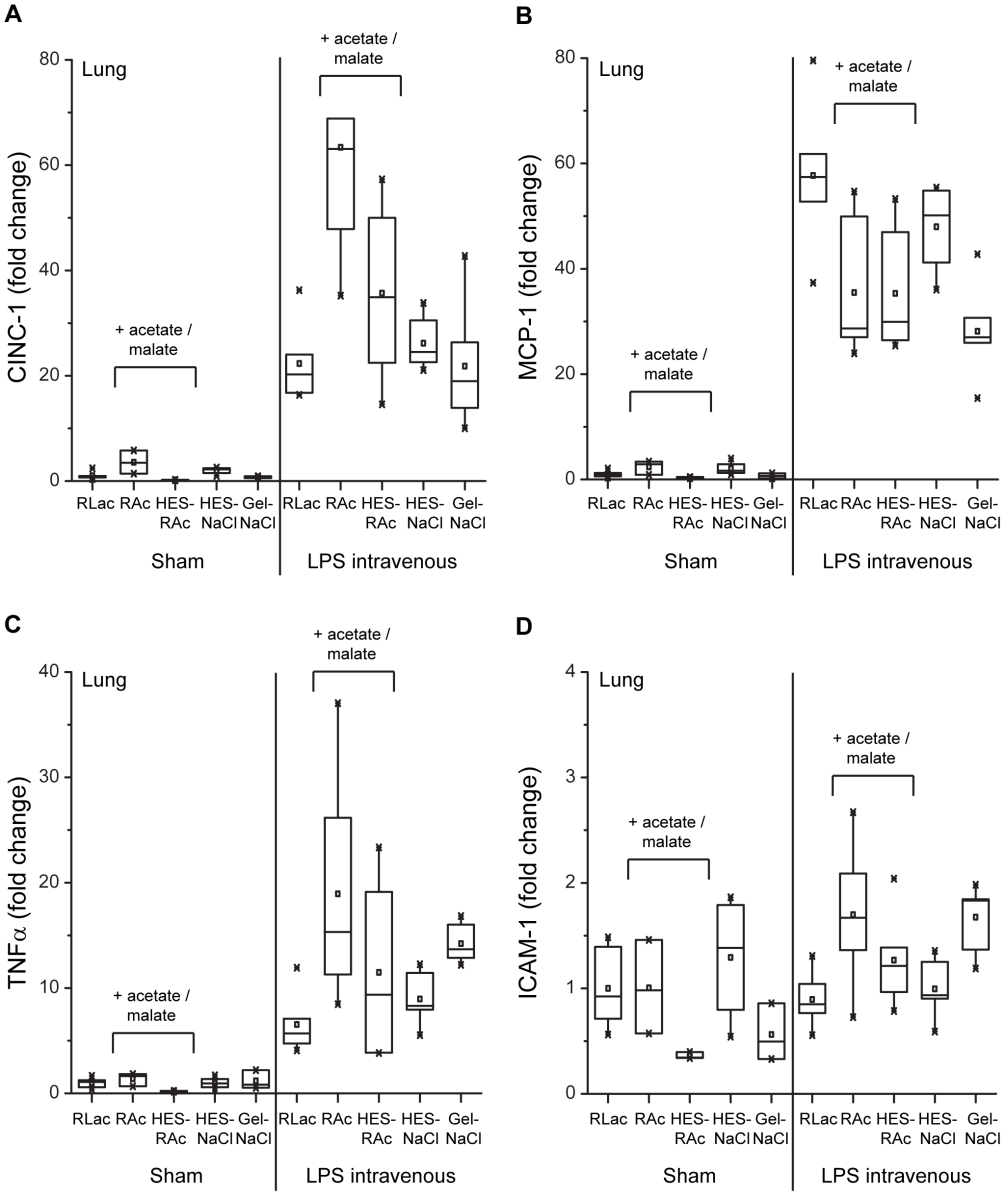
Inflammatory mediator expression in lung tissue. Evaluation of cytokine-induced neutrophil chemoattractant-1 (CINC-1; A), monocyte chemoattractant protein-1 (MCP-1; B), tumor necrosis factor α (TNFα; C) and intercellular adhesion molecule 1 (ICAM-1; D) messenger RNA expression in lung tissue. Lung tissue was collected after 4 hours from the 10 study groups: SHAM (+PBS) or LPS intravenous (+LPS): RLac, HES-NaCl, RAc, HES-RAc, Gel-NaCl. Specific real-time polymerase chain reactions were performed on random transcribed complementary DNA. Values are illustrated as fold change in relation to the RLac group ( = 1.0).

An increase of inflammatory mediator transcripts in lung tissue was measured in LPS-stimulated animals (CINC-1: 32-fold, MCP-1: 41-fold, TNFα: 11-fold, ICAM-1: 0.4-fold). Again HES, independently from the buffering agent, was not measured to have a significant influence on inflammatory mediator expression (**Figure 4A–D** **,** **Table 4**). No effects of gelatin on mRNA levels of inflammatory mediators were observed, except for a reduction by a 17-fold of MCP-1. The use of acetate-buffered solutions was associated with elevated levels of CINC-1 (16-fold) and TNFα (5-fold), while an attenuating effect on MCP-1 expression was measured (10-fold lower expression).

**Table 4:** Changes in inflammatory mediator expression of the lung.

	LPS	HES	Gelatin	Acetate buffer	R^2^
CINC-1, n-fold	**32 (23, 40)^a^**	−1 (−17, 1)	−6 (−18, 5)	**16 (7, 25)^a^**	0.652
MCP-1, n-fold	**41 (35, 47)^a^**	−2 (9, 4)	**−17 (−25, −8)^c^**	**−10 (−17, −4)^b^**	0.815
TNF-α, n-fold	**11 (7, 14)^a^**	−2 (−5, 16)	3 (−2, 8)	**5 (1, 8)^c^**	0.561
ICAM-1, n-fold	**0.4 (0.1, 0.7)^c^**	−0.2 (−0.5, 0.2)	0.2 (−0.3, 0.6)	0.2 (−0.2, 0.5)	0.189

The table shows B coefficients (95% confidence intervals) of the linear regression. Animals which received solely Ringers' lactate were used as reference group. mRNA expression is expressed as a fold-difference relative to the reference group. The different fluid ingredients (HES, gelatin, and acetate buffer) were entered as binary independent predictors in the regression model. Significance: ^a^p≤0.001, ^b^p≤0.01, ^c^p<0.05; CINC-1  =  cytokine-induced neutrophil chemoattractant-1; MCP-1  =  monocyte chemoattractant protein-1; TNFα  =  tumor necrosis factor α; ICAM-1  =  intercellular adhesion molecule-1.

### 2. Acute kidney injury

#### Neutrophil gelatinase associated lipocalin (NGAL)

**Figure 5:**
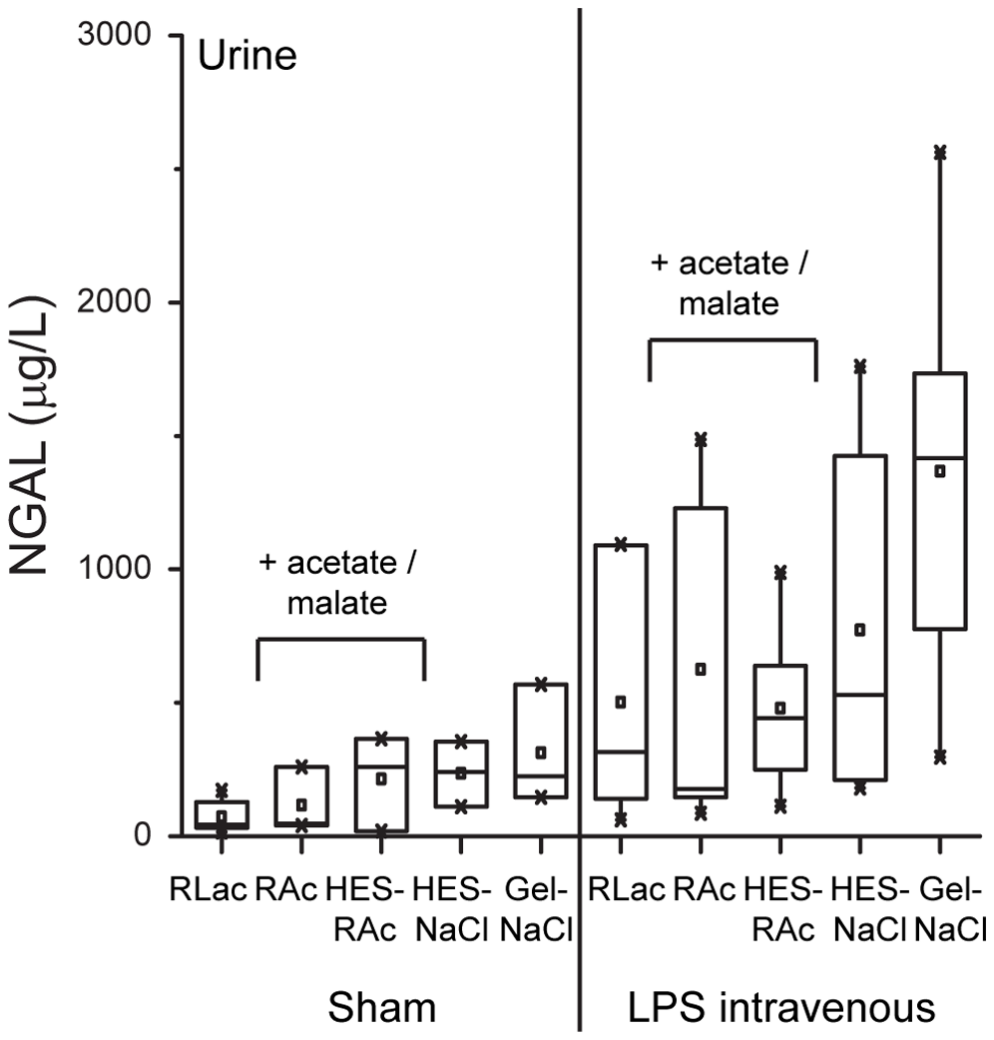
Evaluation of Neutrophil gelatinase-associated lipocalin (NGAL) protein expression in urine. Urine was taken after 4: SHAM (+PBS) or LPS intravenous (+LPS): RLac, HES-NaCl, RAc, HES-RAc, Gel-NaCl. Specific enzyme-linked immunosorbent assays were performed with urine.

For evaluation of acute kidney injury, NGAL mRNA expression in the kidneys, as well as NGAL protein concentrations in urine were measured at the end of the experiment (at 4 hours). Changes in expression of NGAL mRNA were below a difference of a 1-fold, independent of stimulation with LPS and independent of the type of fluid (**Table S2**). In urine, concentration of NGAL protein was increased by +555 μg/L in response to LPS stimulation (p≤0.001, **Table S2**). HES had no significant influence on NGAL protein levels. Gelatin, however, provoked an increase in NGAL protein levels by +582 μg/L (p≤0.01). No influences of solutions containing an acetate buffer were observed on NGAL protein levels (**Figure 5**).

### 3. Mean arterial blood pressure

Mean arterial blood pressure from LPS-stimulated animals did not differ from animals, which received PBS instead of LPS (p = 0.183, R^2^: 0.177). The different types of fluid (crystalloids or colloids) used for fluid resuscitation had no influence on MAP (p = 0.370, R^2^: 0.143).

### 4. Blood gas analysis

Stimulation with LPS decreased pH (Δ-0.69, p = 0.003, R^2^:0.320) and hydrogencarbonate (HCO_3-_) levels (Δ-1.2 mmol/L, p = 0.021, R^2^: 0.284), while partial pressures of carbon dioxide (pCO_2_) in blood increased by +0.8 kPa (p = 0.013, R^2^: 0.429). No influence on lactate levels, oxygen partial pressures (pO_2_), or electrolytes was observed. Fluid resuscitation with a HES-containing fluid was associated with a decrease in pH (Δ-0.085, p<0.001, R^2^:0.320) and an increase in pCO_2_ partial pressures (Δ+1.0 kPa, p = 0.001, R^2^: 0.429). Electrolytes did not change after HES application. Fluid resuscitation with gelatin only influenced pO_2_ (Δ-5.2 kPa, p = 0.014, R^2^: 0.669), potassium levels (Δ-0.8 mmol/L, p<0.001, R^2^: 0.582), and chloride levels (Δ+3.5 mmol/L; p = 0.024, R^2^: 0.268). Application of both colloids – HES and gelatin – was associated with lower serum lactate levels (p = 0.004, R^2^: 0.174). Administration of acetate-buffered solutions did not influence pH, HCO_3-_, and lactate concentrations. However, pCO_2_ (Δ-0.9 kPa, p = 0.009, R^2^: 0.429) and pO_2_ were slightly lower in animals receiving acetate-buffered solutions (Δ-5.1 kPa, p = 0.003, R^2^: 0.669). Also sodium (Δ -1.8 mmol/L, p = 0.027, R^2^: 0.468) and potassium concentrations (Δ -0.5 mmol/L, p = 0.003, R^2^: 0.582) were marginally decreased by acetate-buffered solutions, while chloride levels remained unchanged. No difference in serum lactate levels were measured when resuscitation was performed using RLac compared to lactate-free solutions (p = 0.184, R^2^: 0.174).

## Discussion

The present study compared inflammatory and haemodynamic effects of different saline-based or buffered, plasma adapted crystalloid and colloidal solutions in a rodent model of LPS-induced inflammation in rats. The key finding of the study is that acetate-buffered solutions induce a significant higher inflammatory response in the kidney, the liver and the lung as measured by expression of CINC-1 and TNFα in rats suffering from acute endotoxemia.

### Impact of HES solutions

Several animal studies have evaluated the impact of different HES preparations on renal function in sepsis. In a 24-hour CLP model in rats Schick and colleagues found higher serum level of the early kidney injury marker NGAL when fluid resuscitation was performed with colloids (gelatin 4% or HES 6% 130/0.42), whereas the balanced crystalloid Sterofundin ISO caused the least effects on kidney function [16]. Similar to our study no significant differences in vital parameters such as MAP, heart rate and oxygenation were observed among the different fluid management groups. However, this study did not differentiate between “balanced” HES and “non-balanced” HES as only saline-based HES preparations were studied.

In a recent study of Simon et al., the effect of balanced HES 130/0.42, balanced gelatin, RAc and the older HES 200/0.5 in saline was tested in a 19-hour two hit model consisting of haemorrhagic and septic shock [17]. Haemodynamic parameters (cardiac output, MAP, central venous pressure) did not differ significantly between groups 12 hours after induction of sepsis. Inflammatory response of the kidney (measured by interleukin, IL-6, TNFα and IL-10) as well as parameters of renal function (serum creatinine, creatinine-clearance) were significantly elevated in the 10% HES 200/0.5 group. Again the study compared different HES preparations in different solutions (HES 200/0.5 in saline versus HES 130/0.42 in RAc) which complicates a differentiation between “HES-induced” and “solvent-induced effects”.

### Impact of acetate-buffered solutions

The concept of fluid resuscitation with balanced solutions containing acetate (+ malate) is relatively new. The impact of a fluid resuscitation with these balanced solutions – no matter if colloid or crystalloid – on the clinical outcome especially in a septic status has not yet been determined [14].

Aksu and colleagues assessed the effects of unbalanced HES (HES in NaCl) and balanced HES (HES in RAc) on kidney function in a model of LPS-induced septic shock in rats [18]. They found that both solutions improve MAP and could normalize creatinine clearance. Only HES-RAc could increase renal microvascular perfusion. None of the fluids could improve metabolic acidosis and plasma ion levels.

A previous study of the group of Ertmer explored the effect of unbalanced and balanced HES in comparison to a balanced crystalloid in a model of endotoxemia in sheeps. Higher plasma creatinine and urea concentrations in both HES groups were found compared to the crystalloid group. Renal function measured by creatinine clearance and cumulative creatinine excretion were similar in all septic groups. Moreover, electron microscopy showed less renal tubular injury and a higher percentage of intact microvilli brush borders in the HES groups compared to the balanced crystalloid group. The author concludes that renal function and ultrastructural integrity is preserved with the use of 6% HES solutions [19].

The key finding of our study is that acetate-buffered solutions provoke a higher inflammatory response in endotoxemia independently if a colloid or crystalloid was used. One possible explanation could be that the chemical composition of the buffering agent, especially of the organic anions, influences the inflammatory response: Our balanced solutions always contained 24 mmol/l of acetate and 5 mmol/l of malate as a buffer, whereas in the cited studies only balanced solutions containing acetate were used. Interestingly, no increase in inflammatory response was detected when Ringers' Lactate (also a balanced solution containing lactate as organic buffer) was infused for fluid resuscitation.

### Possible adverse effects of acetate and malate

The effect of acetate in the vasculature has been studied extensively in the past. In renal replacement therapy it has been shown that acetate can have pro-inflammatory, myocardial depressant and hypoxemia promoting properties [20], [21]. Therefore, it has been removed from fluids currently used for renal replacement therapy.

Davies et al. compared an acetate/gluconate-based priming solution with a bicarbonate-based one for cardio-pulmonary bypass and could demonstrate persistent supra-physiological acetate and gluconate levels upon the use of acetate/gluconate-based solution. Although IL-6 levels did not differ significantly in this study the author concluded that one cannot rule out that these supra-physiological anion levels can be harmful [22]. Acetate has been shown to stimulate cytokine release such as IL-1, to cause carbohydrate intolerance and disturbance of fatty acid synthesis [23]. Moreover a direct myocardio-toxic effect has been suggested. Therefore it is convincing that haemodiafiltration using an acetate-free buffer is associated with less deterioration of hemodynamics and myocardial contractility [24].

Malate in combination with α-ketoglutarate has been suggested to have protective properties in ischemia-induced AKI *in vitro* [25]. However, in a recent study the *in vivo* application of malate/α-ketoglutarate in a model of ischemia-induced AKI resulted in a cardiovascular depression without any protective effect on kidney function [26].

In our study we demonstrate that acetate-buffered solutions provoke a significant higher expression of CINC-1 and TNFα in the kidney as well as in the liver and the lung. Therefore, one could hypothesize that a balanced solution containing acetate and malate could be most harmful for an already injured kidney, for example after LPS challenge like in our model or in a septic patient.

### Possible mechanism of inflammatory response: Effects of CINC-1 and TNFα up-regulation

Rat CINC-1 is part of the CXC-chemokine family. It plays a pivotal role in neutrophil-mediated inflammatory diseases by attracting neutrophils to the site of inflammation [27], [28]. TNFα as a pleiotropic cytokine being of high importance in inflammatory kidney diseases has been shown to have pro-inflammatory properties (promoted via the TNFR2-receptor) as well as immuno-regulatory functions (promoted via the TNFR1-receptor) [29].

The main cause of AKI in sepsis is a reduction in general or localized renal blood flow resulting in a generalized or localized ischemia. Ischemic-induced kidney injury involves a complex cellular pathophysiology including a general inflammatory response as well as epithelial [acute tubular necrosis (ATN)] and endothelial cell injury [30].

The damage of the endothelial cell results in a loss of glycocalyx, alterations of cell-cell contacts and increase of vascular permeability resulting in a shift of fluid into the renal interstitium [31]. We hypothesize that application of a balanced solution (with or without HES) in a renal vascular compartment with already injured endothelial cells induces a local accumulation of pro-inflammatory cytokines (e.g. CINC-1), which results in increased recruitment of immune cells like neutrophils and macrophages. These immune cells can cause a direct injury of the tubular epithelial cell. We could clearly demonstrate a significant higher expression of CINC-1 mRNA-levels in kidneys of LPS-treated animals that receive a balanced solution. Interestingly, it has been shown that CINC-1 possess all characteristics of an acute phase protein as it shows an acute and transient increase in protein expression in serum and liver 2 hours after endotoxin application [32]. We were able to show that the expression of CINC-1 mRNA in the liver is significantly more pronounced when a balanced solution is used. Additionally, lung tissue analysis revealed similar expression pattern of CINC-1. Therefore one could hypothesize that the balanced solution could lead to a more pronounced systemic inflammation in an already injured organism.

It has been shown that HES has anti-inflammatory characteristics by attenuating chemotaxis of inflammatory cells and thereby improving pulmonary function during endotoxemia as well as decrease the production of hepatic inflammatory mediators [33]. Interestingly, the HES preparation used in that study was saline-based. Levels of inflammatory markers in our saline-based HES group were comparable low even after LPS challenge. This supports the thesis that buffering agent is a crucial factor in the inflammatory effect of different fluid preparations.

### Acute Kidney Injury: NGAL expression

Neutrophil gelatinase-associated lipocalin is a protein of the lipocalin superfamily that is expressed by proximal tubule cells [34], [35]. It has been shown to be up-regulated very early in post-ischemic kidney of mice and rats and in a nephrotoxic model in mice [36]. Additionally, it is a promising and sensitive biomarker of AKI in human [37]. Recently, it was found that NGAL mRNA up-regulation in tubular epithelial cells is sensitive to LPS-induced AKI in rats. Interestingly the up-regulation occurred within 3 hours after LPS administration and correlated with urinary NGAL levels [38].

Our results revealed an up-regulation of NGAL mRNA expression in the kidney and protein expression in urine 4 hours after the application of LPS. Although NGAL is a very sensitive and early marker of AKI Han et al. could recently demonstrate that the peak of NGAL mRNA and protein expression is found 6 hours after intravenous LPS application [38]. Although we could show that acetate-buffered solutions led to a significant lower expression of NGAL mRNA one could hypothesize that the time point of our measurements was too early in order to reach the maximum mRNA expression.

Serum creatinine is a late marker of impaired kidney function. It increases only after a loss of at least 50% of renal function and requires several hours to days to accumulate [39]. Therefore we did not measure serum creatinine levels in our experiment.

### Limitations

We decided to use a model of LPS-induced endotoxemia in rats: the systemic administration of LPS offers several advantages as it is a highly standardized and reproducible model which induces a quick and acute response [40]. Tough the administration of LPS to a human being induces the clinical manifestations of a septic shock and subsequent organ dysfunctions [41], a single injection of LPS is unlikely to mimic all facets of human sepsis. Important aspects of human sepsis are known to be better mimicked in other animal models of sepsis [42]. Especially, the cytokine profiles, for example, in caecal ligation and puncture models are more likely resembling to those seen in human sepsis [40], [42]. For interrogating systemic and renal responses during the initial phases of sepsis, however, LPS-induced endotoxemia represents a valid tool [42]. As a second point, it has to be taken into account that the duration of our model is very short. Thus, even very early markers of acute kidney injury such as NGAL might not yet be able to detect differences in the present study.

### Conclusion

The study provides evidence that buffering agent of different colloidal and crystalloid solutions could be a crucial factor determining the extent of inflammatory response in sepsis. Thus the use of balanced solutions containing acetate in a septic status may have a higher potential to impair end-organ function due to a more pronounced local inflammatory response.

## Supporting Information

Table S1 **Primers and probes used for the Real-time Quantitative TaqMan PCR.**
(DOCX)

Table S2 **Linear regression on production of neutrophil gelatinase associated lipocalin (NGAL).**
(DOCX)
