# Supplementary material for: Modulation of Early Inflammatory Response by Different Balanced and Non-Balanced Colloids and Crystalloids in a Rodent Model of Endotoxemia
Source: PLoS One. 2014 Apr 7;9(4):e93863. doi: 10.1371/journal.pone.0093863 (PMC3977866; doi:10.1371/journal.pone.0093863)
Supplement: Table S1 — Primers and probes used for the Real-time Quantitative TaqMan PCR. (DOCX) [file pone.0093863.s001.docx]

***Table S1. Primers and probes used for the Real-time Quantitative TaqMan PCR****

| ***Gen*** | ***Primer Sequence*** | ***Length of Amplicon (nt)*** |
| --- | --- | --- |
| **CINC-1**  Up  Down  *Probe # 49* | 5’ CAC ACT CCA ACA GAG CAC CA 3’  5’ TGA CAG CGC AGC TCA TTG 3’  5’ CAG CCA CC 3’ | 120 |
| **MCP-1**  Up  Down  *Probe # 62* | 5’ AGC ATC CAC GTG CTG TCT C 3’  5’ GAT CAT CTT GCC AGT GAA TGA GT 3’  5’ ACC TGC TG 3’ | 78 |
| **TNFα**  Up  Down  *Probe # 49* | 5’ GCC CAG ACC CTC ACA CTC 3’  5’ GAG CCC ATT TGG GAA CTT CT 3’  5’ CAG CCA CC 3’ | 99 |
| **ICAM-1**  Up  Down  *Probe # 74* | 5’ TTC TGC CAC CAT CAC TGT GT 3’  5’ AGC GCA GGA TGA GGT TCT T 3’  5’ GGC AGC AG 3’ | 95 |
| **NGAL**  Up  Down  *Probe # 1* | 5’ CAC TTC CCT CGT CAG G 3’  5’ AAT ATT CCC CAG GGT GAA CTG 3’  5’ GCT CCA GG 3’ | 94 |
| **18S**  Up  Down  *Probe # 74* | 5’ GGA GCC TGA GAA ACG GCT A 3’  5’ TCG GGA GTG GGT AAT TTG C 3’  5’ GGC AGC AG 3’ | 64 |

* P.E. Applied Biosystems, Waltham, MA; CINC-1 = cytokine-induced neutrophil chemoattractant-1; MCP-1 = monocyte chemoattractant protein-1; TNFα = tumor necrosis factor α; ICAM-1 = intercellular adhesion molecule-1; NGAL = neutrophil gelatinase-associated lipocalin; 18S = housekeeping gene; nt = nucleotides
