# Supplementary material for: Modulation of Early Inflammatory Response by Different Balanced and Non-Balanced Colloids and Crystalloids in a Rodent Model of Endotoxemia
Source: PLoS One. 2014 Apr 7;9(4):e93863. doi: 10.1371/journal.pone.0093863 (PMC3977866; doi:10.1371/journal.pone.0093863)
Supplement: Table S2 — Linear regression on production of neutrophil gelatinase associated lipocalin (NGAL). (DOCX) [file pone.0093863.s002.docx]

**Table S2. Linear regression on production of neutrophil gelatinase associated lipocalin (NGAL)**

|  | **LPS** | **HES** | **Gelatin** | **Acetate buffer** | **R^2^** |
| --- | --- | --- | --- | --- | --- |
| NGAL, n-fold | **0.4 (0.3, 0.5) ^a^** | 0.0 (-0.2, 0.1) | **-0.2 (-0.4, 0) ^c^** | **-0.2 (-0.3, 0) ^b^** | 0.521 |
| Urine NGAL, µg/L | **555 (259, 851) ^a^** | 87 (-234, 408) | **582 (167, 996) ^b^** | -58 (-379, 263) | 0.386 |

The table shows B coefficients (95% confidence intervals) of the linear regression. Animals which received solely Ringers’ lactate were used as reference group. NGAL mRNA expression is expressed as a fold-difference relative to the reference group. The different fluid ingredients (HES, gelatin, and acetate buffer) were entered as binary independent predictors in the regression model. Significance: ^a^ p≤0.001, ^b^ p≤0.01, ^c^ p<0.05;
